# Asthma and high-intensity interval training have no effect on clustered cardiometabolic risk or arterial stiffness in adolescents

**DOI:** 10.1007/s00421-020-04590-4

**Published:** 2021-03-29

**Authors:** M. A. McNarry, L. Lester, E. A. Ellins, J. P. Halcox, G. Davies, C. O. N. Winn, K. A. Mackintosh

**Affiliations:** 1 Applied Sports, Technology, Exercise and Medicine Research Centre, College of Engineering, Swansea University, Bay Campus, Swansea, SA1 8EN UK; 2 School of Human Sciences, University of Western Australia, Perth, 6009 Australia; 3 Swansea University Medical School, Swansea University, Swansea, SA2 8PP UK

**Keywords:** Augmentation index, Blood lipids, Principal component analysis, Pulse wave velocity, Youth, HIIT

## Abstract

**Purpose:**

Cardiometabolic risk, including arterial stiffness, is increasing in youth. Those with asthma are suggested to be particularly at risk of cardiovascular disease. Efficient and effective strategies are required to prevent the atherosclerotic process in youth. The purpose of this study was to investigate the effect of 6 months high-intensity interval training (HIIT) on cardiometabolic risk in youth with and without asthma.

**Methods:**

65 adolescents (31 mild asthma; 34 non-asthma) were recruited, 32 (16 asthma) of whom were randomly allocated to receive HIIT three times per week for 6 months. At baseline, mid-intervention, post-intervention and at a 3-month follow-up, anthropometric, metabolic and vascular determinants of cardiometabolic risk were assessed. Following principal component analysis (PCA), linear mixed models were used to assess the influence of asthma, HIIT and their interaction.

**Results:**

Seven factors were identified which explained 88% of the common variance shared among the parameters. Those with asthma demonstrated lower arterial stiffness factor scores mid-intervention (*P* = 0.047) and lower cholesterol factor scores post-intervention (*P* = 0.022) but there was no effect of the intervention, or interaction effects, on any PCA-identified factor, at any time-point. HIIT was associated with a lower low-density lipoprotein and diastolic blood pressure at mid-intervention.

**Discussion:**

Neither arterial stiffness nor clustered cardiometabolic risk are influenced by HIIT in adolescents with or without asthma, despite important changes in blood lipid and pressure profiles. Blood pressure, augmentation and pulse wave velocity should be considered physiologically distinct constructs and as potential markers of cardiovascular health.

## Introduction

Asthma, a chronic inflammatory disorder of the airways associated with airway obstruction and hyper-responsiveness, affects 1 in 11 children in the UK (Asthma UK 2018). However, the inflammation that occurs in those with asthma is not restricted to the lungs, with elevated pro-inflammatory cytokines leading to systemic inflammation (Wood et al. 2012; Girdhar et al. 2011), and potentially the higher rate of cardiovascular disease epidemiologically observed in adults with asthma (Onufrak et al. 2007; Schanen et al. 2005; Iribarren et al. 2012). The underlying atherosclerotic process is often initiated during childhood (McGill et al. 2000; Hong 2010) and is exacerbated and accelerated by the presence of cardiometabolic risk factors (Katzmarzyk et al. 2004; McGill et al. 2002; Zimmet et al. 2007). Given these risk factors are increasingly evident in youth (Owens 2013), there is a need for primary prevention and intervention strategies that target such modifiable risk factors during childhood (Hong 2010).

Cardiorespiratory fitness and exercise are imperative for the management of asthma; exercise is associated with improved lung function and mental health (Avallone and McLeish 2013), as well as helping to prevent, or at least diminish, the symptoms of asthma (Andrade et al. 2014; Westergren et al. 2016). Furthermore, exercise is suggested to be cardioprotective from the development of atherosclerosis and subsequent cardiovascular disease (Thompson et al. 2003). However, interventions seeking to improve cardiometabolic health have had varied success.

High-intensity interval training (HIIT) provides a potent stimulus to cardiorespiratory fitness and cardiometabolic health in youth (Logan et al. 2014; Costigan et al. 2015; Eddolls et al. 2017). Potentially beneficial effects of HIIT have also been reported for body composition (Westergren et al. 2016; Bailey et al. 1995) and some, although not all (Aparecido da Silva et al. 2016), have suggested that HIIT is less likely to induce bronchoconstriction (O'Neill et al. 2017; Good et al. 2019). However, the generalizability of these findings to a real-world setting is limited by a reliance on laboratory-based protocols, relatively short interventions and a lack of follow-up (Costigan et al. 2015; Eddolls et al. 2017; Logan et al. 2014). Weston et al. (2016) developed a multi-activity, low-volume, high-intensity games intervention that was delivered in a school-setting, reporting generally smaller, but nonetheless clinically meaningful, changes in triglycerides (TG), waist circumference, moderate-to-vigorous physical activity (MVPA) and estimated cardiorespiratory fitness. However, a methodological limitation of this, and, indeed, many earlier studies, is their focus on the components of cardiometabolic risk as individual factors, despite considerable evidence of the clustering of cardiometabolic risk factors (Reaven 1988). Indeed, one factor is unlikely to define cardiometabolic health in youth (Stoner et al. 2017) and thus influencing individual factors does not necessarily translate to beneficial changes in the overall risk score. Nonetheless, the specific composition of the clustered cardiometabolic risk score in youth continues to be a contentious issue, with recent evidence suggesting that central blood pressure and measures of arterial stiffness should be included (Stoner et al. 2017).

Arterial stiffness is suggested to represent a powerful prognostic tool (Adji et al. 2011), providing an insight to the hemodynamic impact of adverse structural changes in the arterial system. Asthma is increasingly reported to be associated with a higher arterial stiffness in both children (Steinmann et al. 2015; Augusto et al. 2017), although it is pertinent to note that not all studies have reported such an association (Gulen et al. 2015; Özkan et al. 2016). These equivocal findings may reflect the wide age, and thus maturity, and disease severity ranges within these studies; endothelial dysfunction increases during puberty (Augusto et al. 2017) and is inversely related to lung function or other ratings of disease severity (Steinmann et al. 2015; Sun et al. 2014; Tuleta et al. 2017). Traditional aerobic exercise is associated with significant improvements in arterial stiffness in children (Farpour-Lambert et al. 2009; Meyer et al. 2006; Watts et al. 2004), but the potential utility of HIIT largely remains to be elucidated. Specifically, in the only study to date, Chuensiri et al. (2018) reported improvements in arterial stiffness and flow-mediated dilation in obese pre-pubertal boys following a 3-month cycle ergometer-based HIIT intervention. However, given the age-related changes in pulse wave velocity (PWV; Reusz et al. 2010; Fischer et al. 2012; Thurn et al. 2015), the potential presence of sex differences (Fischer et al. 2012; Thurn et al. 2015; Curcio et al. 2016), and the lack of consideration of those with asthma, further research is required to ascertain whether HIIT is effective at improving arterial stiffness in both healthy adolescents and those with asthma.

Therefore, the purpose of the present study was to determine the influence of asthma on the clustering of cardiometabolic risk factors in youth and to investigate the influence of a 6-month, school-based HIIT intervention on this clustered cardiometabolic risk in adolescents with asthma and their healthy age- and sex-matched counterparts. It was hypothesized that those with asthma would be characterized by a greater cardiometabolic risk and that the HIIT intervention would lead to significant improvements in clustered cardiometabolic risk, irrespective of asthma status.

## Methods

### Participants

A sub-sample of 69 adolescents (13.6 ± 0.9 years; 39 boys; 36 with asthma) were selected to take part in additional lab-based testing using stratified randomization in Microsoft Excel from 616 participants involved in a larger randomized control trial for which participants were recruited from local secondary schools (The X4A trial: eXercise for Asthma with Commando Joe’s). Sixty-five adolescents provided the measures at all 4 time-points and were included in this analysis (13.5 ± 0.9 years; 37 boys; 33 asthma; Table 1), 32 (19 boys; 16 asthma) of whom were randomly allocated to receive a 6-month intervention. Asthma severity was assessed using the Global Initiative for Asthma guidelines (Global Initiative for Asthma 2017) and classified according to the medication step required to achieve asthma control. Participants were excluded if they did not have stable asthma, defined as having experienced an exacerbation within the last 4 weeks. Ethical approval was granted by the institutional research ethics committee (ref: 140515 and PG/2014/29). Parent/guardian consent and child assent were obtained prior to participation. Participants in the control group (33 adolescents; 20 boys; 17 asthma) were asked to continue with their routine activities.

**Table 1:** Baseline participant characteristics according to asthma status and exercise group

	Overall	Asthma		Healthy	
		Intervention	Control	Intervention	Control
Sample size	65	16	17	16	16
Height (cm)	159.4 ± 9.3	161.4 ± 11.2	160.5 ± 9.4	157.5 ± 8.3	158.4 ± 8.4
Weight (kg)	54.1 ± 13.5	58.2 ± 15.0	57.1 ± 14.7	51.2 ± 13.4	50.1 ± 9.6
BMI (kg·m^−2^)	21.1 ± 3.3	22.1 ± 3.7	22.0 ± 4.2	20.3 ± 3.4	19.8 ± 2.8
WC (cm)	69.6 ± 10.0	70.9 ± 11.3	72.4 ± 12.0	68.1 ± 8.6	66.8 ± 7.1
SBP (mmHg)	123 ± 13	122 ± 12	126 ± 14	119 ± 12	126 ± 11
DBP (mmHg)	72 ± 10	72 ± 6	74 ± 12	70 ± 9	74 ± 11
ABP (mmHg)	111 ± 8	113 ± 6	111 ± 11	109 ± 7	110 ± 6
MAP (mmHg)	83 ± 6	84 ± 5	83 ± 6	83 ± 6	82 ± 6
SV (ml)	92.4 ± 16.2	93.3 ± 14.3	94.3 ± 22.9	88.3 ± 10.8	94.6 ± 16.0
HR (bpm)	72 ± 12	72.5 ± 10.0	72 ± 9	73 ± 16	68 ± 11
Q (l·min^−1^)	6.8 ± 1.6	7.0 ± 1.5	7.0 ± 1.9	6.6 ± 1.3	6.6 ± 1.6
TPR (PRU)	0.8 ± 0.2	0.8 ± 0.3	0.8 ± 0.3	0.8 ± 0.1	0.8 ± 0.2
ESP (mmHg)	96.0 ± 9.8	95.3 ± 11.1	96.8 ± 10.3	95.2 ± 8.7	96.9 ± 10.1
SEVR (%)	160.8 ± 60.1	156.5 ± 66.4	167.4 ± 69.6	148.3 ± 34.1	173.4 ± 69.4
PWV (m s^−1^)	5.5 ± 0.6	5.6 ± 0.6	5.6 ± 0.5	5.7 ± 0.7	5.4 ± 0.5
Aug (mmHg)	4.8 ± 3.5	4.7 ± 3.4	4.5 ± 3.2	4.5 ± 3.3	5.5 ± 3.1
AIx (%)	9.2 ± 6.6	9.1 ± 6.4	8.6 ± 4.8	9.0 ± 5.8	10.2 ± 6.0
HDL (mmol·l^−1^)	1.5 ± 0.4	1.3 ± 0.4	1.5 ± 0.4	1.6 ± 0.2	1.5 ± 0.5
ΤC:HDL	2.6 ± 0.6	2.9 ± 0.6	2.7 ± 0.8	2.5 ± 0.4	2.4 ± 0.6
CHO (mmol·l^−1^)	3.6 ± 0.7	3.6 ± 0.7	3.7 ± 0.5	3.7 ± 0.4	3.3 ± 1.1
LDL (mmol·l^−1^)	1.6 ± 0.5	1.7 ± 0.5	1.6 ± 0.7	1.6 ± 0.5	1.6 ± 0.4
TG (mmol·l^−1^)	1.1 ± 0.6	1.1 ± 0.3	1.2 ± 1.1	1.2 ± 0.3	0.9 ± 0.3

Mean ± SD. *ABP* aortic blood pressure, *AIx* augmentation index, *Aug* augmentation, *CHO* cholesterol, *DBP* diastolic blood pressure, *ESP* end systolic pressure, *HDL* high density lipoprotein, *HR* heart rate, *LDL* low-density lipoprotein, *MAP* mean arterial pressure, *PWV* pulse wave velocity, *Q* cardiac output, *SBP* systolic blood pressure, *SEVR* subendocardial viability ratio, *SV* stroke volume, *ΤC* Total Cholesterol, *TG* triglycerides, *TPR* total peripheral resistance, *WC* waist circumference

### Intervention

The intervention design was based on formative work (Winn et al. 2017) and has been reported previously (Winn et al. 2019). Briefly, the intervention consisted of 6 months HIIT, with three 30-min sessions per week consisting of circuits and games-based activities designed to elicit a heart rate of > 90% Heart Rate maximum (HR_max_), with a 1:1 exercise-to-rest ratio. Throughout the intervention, the duration of the exercise bouts was progressively increased from 10 to 30 s. Throughout each session, heart rate (HR) was continuously monitored for each participant (Activio AB, Stockholm, SWE). Maximal HR was predicted using the Tanaka et al. (2001) formula, validated for use in adolescents (Mahon et al. 2010). The intervention was delivered by a trained professional from Commando Joe’s® (Manchester, UK).

Participants were assessed at four time-points baseline, mid-intervention (3 months), post-intervention (6 months) and at a 3-month post-intervention follow-up. Each participant was asked to attend the laboratory at the same time during the school day (± 2 h), in a hydrated state and at least 2 h postprandial.

### Anthropometrics

Stature and sitting stature were measured to the nearest 0.1 cm (Seca213, Hamburg, Germany) and body mass to the nearest 0.1 kg (Seca876, Hamburg, Germany). Waist circumference was measured using a tape measure to the nearest 0.01 m (Seca 203, Hamburg, Germany) and taken at the waist, at the most indented point of the torso between the bottom of the rib cage and the iliac crest. Lower limb length was calculated as the difference between stature and sitting stature and subsequently used to estimate maturity offset using the equations of Mirwald et al. (2002).

### Vascular assessment

Participants were supine with their torso at 35° and rested for five minutes prior to arterial stiffness assessment. The Vicorder (Skidmore Medical Limited, Bristol, UK) was used for pulse wave analysis (PWA) and the measurement of carotid-femoral PWV and hemodynamic variables (cardiac output, stroke volume, total peripheral resistance, heart rate, end systolic pressure, subendocardial viability ratio). For PWA, a Hokanson SC10 cuff was positioned around the right arm. The cuff was inflated to measure blood pressure and then re-inflated to record the brachial artery pressure waveform. From this waveform, augmentation pressure, augmentation index and central blood pressure (alongside other hemodynamic measures) were determined using a transfer function integral to the software. For PWV measurement, a sensor was positioned around the neck and a Hokanson SC10 cuff placed around the thigh. Path length was defined as the distance from the suprasternal notch to the middle of the thigh cuff. Three measures were taken for both PWA and PWV, of which the median values were used for analyses.

### Cardiometabolic risk factors

Peripheral systolic and diastolic blood pressures were measured using the Omron MX3 Plus monitor (Model HEM-742-E; Omron Healthcare UK, Milton Keynes, UK). Measures were taken from participants whilst seated, following at least 5 min rest. A minimum of two readings were obtained, with the mean used for subsequent analysis. Non-fasted blood lipid (total cholesterol (TC), HDL cholesterol and TG) and glucose profiles were determined from fingertip capillary blood samples via the CardioCheck device (Polymer Technology Systems Inc., Indianapolis, USA), a reliable and valid alternative to gold standard methods for cardiovascular risk screening (Gao et al. 2016). In accord with previous research, non-fasted samples were used given that they do not differ to non-fasted samples in a clinically meaningful manner and may be more representative of normal metabolic conditions (Langsted et al. 2008; Sidhu and Naugler 2012). Consequently, low-density lipoprotein cholesterol (LDL) was not utilized in the analysis as the assumption of a constant triglyceride to cholesterol ratio in very low-density lipoprotein particles required for the Friedewald equation is likely to be violated in the non-fasting state (Krishnaveni and Gowda 2015).

### Lung function

Lung function was measured using a portable dry spirometer (Alpha Spirometer, Vitalograph, Buckingham, UK). Each participant was asked to complete three maximal exhalations according to the American Thoracic Society guidelines (1995); exhalations were defined as acceptable if they were all within 5%. Subsequently, the best exhalation was selected and the forced expiratory volume in one second (FEV_1_) and forced vital capacity (FVC) were expressed as a percentage of age, sex and stature specific predicted values (Quanjer et al. 2012).

Fractional exhaled nitric oxide (FeNO) was measured in accordance with the American Thoracic Society guidelines (Dweik et al. 2011) using the NIOX MINO (Aerocrine AB, Solna, Sweden). Visual and audio cues were provided by the computer software throughout each effort, with the final three seconds of exhalation evaluated.

### Statistical analysis

Statistical analysis was conducted in SPSS v 23 (IBM SPSS Statistics Inc., Chicago, IL, USA) and STATA v 15 (StataCorp LP, Texas, USA). Chi-square analysis was used to determine differences in the proportion of participants who had asthma by sex, whilst a Mann–Whitney test was used to determine differences in age by asthma status. Principal component factor analysis (PCA) was conducted to model the interrelationships between variables by identifying the common variance among the variables. Specifically, PCA was used to identify clustered cardiometabolic risk factors from the anthropometric, arterial stiffness and cardiometabolic variables at baseline, with final estimates of communalities iterated from squared multiple item correlations to convergence. Kaiser’s criterion (Eigenvalues >  = 1.0), together with Cattell’s scree test, were used to determine the number of underlying factors. An orthogonal varimax rotation was performed on the principal components and an overall cardiometabolic score was calculated at each time-point by summing the factor scores identified from the baseline data.

Mixed linear regression models within STATA were used to determine the overall effects of time, the intervention and asthma status, and their interactions, on individual and clustered cardiometabolic risk factors and on the overall cardiometabolic score. Given that the interactions of group and asthma status were not significant, these interactions were subsequently removed from all models. A random intercept was included in each model to account for the repeated measures nature of the data, with all models controlling for age, sex and percentage adherence. Pearson’s Correlation Coefficients were used to assess the relationship between lung function and vascular function.

## Results

At baseline, there were no significant differences according to group (control or intervention) or asthma status in anthropometrics, blood lipids, vascular function or hemodynamic parameters (Table 1). Furthermore, there was no significant difference in age (*Z* = − 0.440, *p* = 0.660) or sex (***χ***^*2*^ = 0.843, *p* = 0.359). Those with asthma in the intervention group were characterized as 87% with mild persistent and 13% with moderate/severe asthma; in the control group, those with asthma were categorized as 77 and 23% mild and moderate/severe, respectively. All of those with asthma were taking took short-acting *β*_*2*_ agonists, with 71% of these participants also taking inhaled corticosteroids. There were no differences in lung function between those with asthma in the intervention or control groups (FEV_1%predicted_: 95 ± 21 vs. 96 ± 28%; FVC_%predicted_: 105 ± 13 vs 98 ± 14% predicted; *P* = 0.85 and *P* = 0.11, respectively). There were no adverse events reported during the intervention in either group.

When the parameters of cardiometabolic risk were considered separately (Table 2), a significant intervention effect was evident, with a lower LDL (*β* = 0.4 (0.06–0.68) mmol·l^−1^; *P* = 0.019) in the intervention group at mid-intervention. Furthermore, the intervention group had a lower diastolic blood pressure at mid-intervention (*β* = 4.3 (0.4–8.1) mmHg; *P* = 0.029) and higher subendocardial viability ratio post-intervention (*β* = − 68.7 (−117.6 to −19.8) %; *P* = 0.006) than the control group. Whilst there was a significant effect of time on augmentation index (AIx), which was higher at follow-up (*β* = 3.27 (0.39–6.14) %; *P* = 0.026), there was no effect of the intervention or asthma. In contrast, there was a significant interaction between asthma and time on PWV, which was higher post-intervention in those with asthma (*β* = 0.44 (0.05–0.84) m·s^−1^; *P* = 0.028). There were no correlations between any lung and vascular function parameters at any time-point.

**Table 2:** Participant characteristics at each time-point according to exercise group

	Baseline		Mid-intervention		Post-intervention		Follow-Up	
	Intervention	Control	Intervention	Control	Intervention	Control	Intervention	Control
Height (cm)	159.4 ± 9.8	159.5 ± 8.8	161.3 ± 10.1	161.7 ± 9.0	163.2 ± 10.1	163.0 ± 8.9	165.0 ± 9.8	163.3 ± 9.2
Weight (kg)	54.5 ± 14.4	53.7 ± 12.8	55.3 ± 14.2	54.8 ± 14.9	57.4 ± 15.8	57.0 ± 14.9	59.5 ± 15.6	56.6 ± 15.0
WC (cm)	69.4 ± 9.9	69.7 ± 10.2	69.6 ± 8.8	69.0 ± 10.7	70.2 ± 9.4	69.9 ± 9.4	71.1 ± 9.9	68.7 ± 9.3
SBP (mmHg)	120 ± 8	126 ± 13	123 ± 10	126 ± 13	120 ± 10	121 ± 10	119 ± 10	120 ± 12
DBP (mmHg)	71 ± 5	74 ± 11	72 ± 12	74 ± 11^*^	70 ± 7	68 ± 10	69 ± 8.1	66 ± 8
ABP (mmHg)	111 ± 7	111 ± 8.5	112 ± 7	112 ± 7	110 ± 7	111 ± 9	111 ± 8	111 ± 10
MAP (mmHg)	84 ± 6	82 ± 6	85 ± 5	85 ± 5	82 ± 6	82 ± 6	83 ± 6	83 ± 8
SV (ml)	90.7 ± 12.6	94.4 ± 19.5	91.0 ± 17.3	92.3 ± 15.7	92.1 ± 18.6	95.8 ± 20.2	93.4 ± 16.7	95.2 ± 15.6
HR (bpm)	73 ± 13	70 ± 10	75 ± 15	74 ± 12	68 ± 12	68 ± 11	72 ± 12	69 ± 11
Q (l·min^−1^)	6.8 ± 1.4	6.8 ± 1.8	6.8 ± 2.0	6.8 ± 1.7	6.3 ± 1.7	6.9 ± 1.7	6.9 ± 1.6	6.8 ± 1.5
TPR (PRU)	0.8 ± 0.2	0.8 ± 0.3	0.8 ± 0.3	0.8 ± 0.3	0.8 ± 0.3	0.8 ± 0.2	0.8 ± 0.2	0.8 ± 0.2
ESP (mmHg)	95.3 ± 9.7	96.8 ± 10.0	97.7 ± 10.3	98.0 ± 10.5	96.9 ± 10.2	94.2 ± 11.5	93.3 ± 13.8	93.9 ± 11.2
SEVR (%)	152.2 ± 51.1	170.3 ± 68.3	168.3 ± 71.8	163.6 ± 58.8	188.5 ± 80.1	161.6 ± 65.9^*^	148.6 ± 65.8	152.9 ± 52.8
PWV (m s^−1^)	5.6 ± 0.6	5.5 ± 0.5	5.7 ± 0.4	5.6 ± 0.3	5.6 ± 0.6	5.5 ± 0.5	5.7 ± 0.6	5.6 ± 0.6
Aug (mmHg)	5.3 ± 3.0	5.4 ± 3.6	4.4 ± 3.0	3.9 ± 3.0	4.3 ± 2.3	5.5 ± 3.5	6.5 ± 3.4	5.3 + 3.6
AIx (%)	9.3 ± 5.9	9.7 ± 7.2	8.6 ± 5.4	7.6 ± 5.6	7.9 ± 4.3	10.4 ± 6.2	12.4 ± 5.7	10.0 ± 6.3
HDL (mmol·l^−1^)	1.1 ± 0.3	1.5 ± 0.4	1.4 ± 0.3	1.5 ± 0.3	1.6 ± 0.4	1.4 ± 0.5	1.4 ± 0.3	1.3 ± 0.3
ΤC:HDL	2.7 ± 0.6	2.6 ± 0.7	2.5 ± 0.6	2.6 ± 0.6	2.4 ± 0.7	2.5 ± 0.6	2.7 ± 0.7	2.6 ± 0.8
CHO (mmol·l^−1^)	3.6 ± 0.6	3.5 ± 0.9	3.4 ± 0.5	3.7 ± 0.5	3.6 ± 0.6	3.6 ± 0.6	3.5 ± 0.5	3.5 ± 0.4
LDL (mmol·l^−1^)	1.7 ± 0.5	1.6 ± 0.5	1.5 ± 0.5	1.7 ± 0.4^*^	1.6 ± 0.5	1.6 ± 0.5	1.7 ± 0.4	1.5 ± 0.6
TG (mmol·l^−1^)	1.1 ± 0.3	1.1 ± 0.8	1.1 ± 0.6	1.1 ± 0.6	1.0 ± 0.5	1.1 ± 0.9	1.1 ± 0.5	1.1 ± 0.6

Mean ± SD. *ABP* aortic blood pressure, *AIx*, augmentation index, *Aug* augmentation, *CHO* cholesterol, *DBP* diastolic blood pressure, *ESP* end systolic pressure, *HDL* high density lipoprotein, *HR* heart rate, *LDL* low-density lipoprotein, *MAP* mean arterial pressure, *PWV* pulse wave velocity, *Q* cardiac output, *SBP* systolic blood pressure, *SEVR* subendocardial viability ratio, *SV* stroke volume, *ΤC* Total cholesterol, *TG* triglycerides, *TPR* total peripheral resistance, *WC* waist circumference

*Significant difference between intervention and control within time-point, *P* < 0.05

The principal component analysis extracted seven factors from the baseline data, accounting for 88% of the common variance between parameters, with factor loadings ranging from 0.44 to 0.95 (Table 3). The seven factors were labeled: a blood pressure/stroke volume factor, an adiposity factor, a hemodynamic/perfusion factor, a hemodynamic/arterial stiffness factor, a cholesterol factor, a wave reflection factor and a TG factor. The summed risk scores for each factor are shown in Tables 4 and 5 according to asthma and intervention group, respectively. After taking into account age and sex, the results indicate there were no statistically significant differences between summed factor scores according to time-point or asthma status for the blood pressure/stroke volume, adiposity, hemodynamic/perfusion, wave reflection or TG factors (all *P* > 0.05). Participants with asthma had significantly lower hemodynamic/arterial stiffness scores mid-intervention (*β* = − 8.87 (confidence intervals: − 17.70–− 0.04); *P* = 0.049) and lower cholesterol scores at post-intervention (*β* = 0.60 (confidence intervals: 0.07–1.13); *P* = 0.028), compared to healthy controls. There was no effect of the intervention, or interaction between asthma and the intervention, for any of the other factors at any time-point.

**Table 3:** Factor loadings of the principal component analysis

	Blood pressure/Stroke volume	Adiposity	Haemodynamic/Perfusion	Haemodynamic/Arterial Stiffness	Cholesterol	Wave reflection	Triglycerides
SBP	0.794	–	–	–	–	–	–
Aortic PP	0.947	–	–	–	–	–	–
Aortic BP	0.769	–	–	–	–	–	–
PP	0.922	–	–	–	–	–	–
SV	0.757	–	–	–	–	–	–
Height	–	0.730	–	–	–	–	–
Weight	–	0.909	–	–	–	–	–
WC	–	0.867	–	–	–	–	–
HDL	–	− 0.851	–	–	–	–	–
TC/HDL	–	0.795	–	–	–	–	–
Q	–	–	− 0.754	–	–	–	–
TPR	–	–	0.894	–	–	–	–
ESP	–	–	0.709	–	–	–	–
SEVR	–	–	0.949	–	–	–	–
MAP	–	–	–	0.912	–	–	–
HR	–	–	–	0.642	–	–	–
PWV	–	–	–	0.442	–	–	–
DBP	–	–	–	0.865	–	–	–
CHO	–	–	–	–	0.980	–	–
LDL	–	–	–	–	0.977	–	–
Aug	–	–	–	–	–	0.783	–
Aix	–	–	–	–	–	0.952	–
TG	–	–	–	–	–	–	0.879
Eigenvalue	5.6	3.8	3.2	2.4	2.3	1.7	1.1
% VE	24.3	16.7	14.1	10.4	10.0	7.2	4.9
% CV	24.3	41.0	55.1	65.5	75.5	82.8	87.6

*ABP* aortic blood pressure, *AIx* augmentation index, *Aug* augmentation, *CHO* cholesterol, *CV* cumulative variance, *DBP* diastolic blood pressure, *ESP* end systolic pressure, *HDL* high density lipoprotein, *HR* heart rate, *LDL* low-density lipoprotein, *MAP* mean arterial pressure, *PWV* pulse wave velocity, *Q* cardiac output, *SBP* systolic blood pressure, *SEVR* subendocardial viability ratio, *SV* stroke volume, *ΤC* Total cholesterol, *TG* triglycerides, *TPR* total peripheral resistance, *VE* variance explained, *WC* waist circumference

**Table 4:** Mean values for PCA-identified factors according to time and asthma status

	Baseline		Mid-intervention		Post-intervention		Follow-up	
	Healthy	Asthma	Healthy	Asthma	Healthy	Asthma	Healthy	Asthma
Blood pressure/SV	426.4	440.6	430.6	434.0	431.2	438.4	432.6	432.5
Adiposity	279.7	294.0	272.6	295.7	286.3	300.9	287.8	304.1
Haemodynamic/Perfusion	263.0	265.8	275.8	262.9	281.7	271.1	234.1	257.7
Hemodynamic/Arterial Stiffness	218.3	222.1	225.4	217.1	215.1	210.0	217.5	214.8
Cholesterol	5.3	5.2	5.2	5.1	4.8	5.3	5.0	5.1
Wave reflection	14.2	13.4	12.7	10.9	14.0	12.8	17.5	16.4
Triglycerides	1.0	1.2	0.9	1.2	1.0	1.1	1.1	1.1
Total	1178.8	1211.2	1099.3	1127.1	1079.1	1128.8	1057.8	1107.8

*PWV* pulse wave velocity, *SV* stroke volume

**Table 5:** Mean values for PCA identified factors according to time and group

	Baseline		Mid-intervention		Post-intervention		Follow-up	
	Intervention	Control	Intervention	Control	Intervention	Control	Intervention	Control
Blood pressure/SV	428.5	438.7	433.6	431.0	435.4	434.2	433.0	432.1
Adiposity	287.1	286.3	289.8	277.9	294.2	291.7	299.0	290.6
Haemodynamic/Perfusion	255.0	274.7	273.6	265.1	292.5	260.3	249.5	251.4
Hemodynamic/Arterial Stiffness	223.6	216.4	222.9	219.7	214.2	211.0	220.0	211.9
Cholesterol	5.3	5.1	4.9	5.3	5.1	5.1	5.0	5.0
Wave reflection	13.6	14.1	12.6	11.0	11.6	15.3	18.2	15.6
Triglycerides	1.1	1.1	1.1	1.1	1.0	1.1	1.1	1.1
Total	1213.8	1175.0	1131.1	1044.3	1105.8	1051.3	1081.3	1031.4

*PWV* pulse wave velocity, *SV* stroke volume

There was no association between markers of lung function and those of arterial stiffness at baseline or mid-intervention but, at post-intervention, FeNO was inversely associated with augmentation (*r* = − 0.42, *P* = 0.029) and augmentation index (*r* = − 0.40, *P* = 0.039). There was no relationship between arterial stiffness markers and the individual markers of cardiometabolic risk.

## Discussion

The main findings of this study were that the cardiometabolic health of adolescents can be almost entirely explained (88%) by seven factors identified by principal component analysis and that the clustering of these risk factors is not dependent on asthma status. The seven factors identified were blood pressure/stroke volume, adiposity, hemodynamic/perfusion, hemodynamic/arterial stiffness, cholesterol, wave reflection and TGs. Furthermore, in the longest school-based HIIT intervention to date, we observed no changes in clustered cardiometabolic risk throughout the intervention period, despite the intervention being associated with an improved blood lipid and blood pressure profile mid-intervention. Importantly, asthma status did not influence the cardiometabolic response to the HIIT intervention.

Asthma is characterized by fluctuating airway and systemic inflammation (Expert Panel Report 3,2007), with chronic inflammation suggested to be associated with increased arterial stiffness and, subsequently, cardiovascular morbidity (Tousoulis et al. 2011; Labat et al. 2013). Indeed, it has previously been reported that children with asthma may demonstrate a higher PWV than their healthy counterparts (Steinmann et al. 2015) and an increased carotid intima media thickness (Dratva et al. 2018). In contrast, the present findings demonstrate no influence of asthma on markers of arterial stiffness and, in agreement with Augusto et al. (2017), neither FeNO nor lung function were associated with arterial stiffness, except at post-intervention. These discordant findings may reflect physiological and/or methodological factors. Specifically, inter-study differences in the severity or control of asthma within the participants could, at least in part, explain these findings, with the adolescents in the current study characterized by relatively mild, well-controlled asthma who may be anticipated to have minimal derangements in vascular function. Indeed, it is feasible that the influence of asthma on arterial stiffness in the present study may have been masked by a mis-diagnosis of asthma or by a self-selection bias amongst the adolescents with asthma due to the exercise-based nature of this study. However, it is also pertinent to note the male bias within Steinmann et al. (2015) given that boys with asthma are suggested to be more at risk of early vascular changes. Further conclusions regarding these discrepancies are limited by inter-study differences in the devices used to determine PWV and in the estimation of pulse wave travel distances (Weber et al. 2009). Longitudinal studies that incorporate a range of disease severities and account for the concomitant influences of age, maturation, sex and fitness are needed to further elucidate the influence of asthma on the arterial stiffness, and thus cardiovascular morbidity risk, of adolescents.

It is suggested that cardiovascular and metabolic disease risk may be increased in those with asthma due to elevated systematic inflammatory mediators (Uzunlulu et al. 2011). However, few have investigated the influence of asthma on cardiometabolic health, with the majority of those that have concentrating on the concomitant and potentially synergistic effects of obesity and asthma (Forno et al. 2015). The current findings regarding a similar clustering of cardiometabolic risk factors irrespective of asthma status in normal weight youth are therefore of interest. Indeed, our findings are in accord with previous exploratory factor analysis studies in children that have consistently highlighted that a single factor cannot explain the cardiometabolic health of youth (Goodman et al. 2009; Stoner et al. 2017; Lambert et al. 2004). However, in contrast to the two-four factors most commonly reported elsewhere (Goodman et al. 2009; Stoner et al. 2017; Lambert et al. 2004), we identified seven factors that explained 88% of the variation in cardiometabolic health in the current adolescents. Whilst this greater number of factors may simply be consequent to a larger range of factors and types of risks considered in the present study (Meigs 2000), the heterogeneity in the factors identified may be indicative of separate metabolic processes rather than a single, unifying pathogenic phenomenon (Hanson et al. 2002; Lindblad et al. 2001). Indeed, it is interesting to note the loading of AIx and PWV onto separate factors in the present study, both of which were also independent of blood pressure. An increased arterial stiffness is associated with a greater PWV, resulting in the reflected wave arriving back at the ascending aorta during systole, augmenting aortic (central) blood pressure. Despite the interdependence of this process, the findings of this study support those of Stoner et al. (2017) that AIx and blood pressure represent different underlying physiological constructs, and extends this conclusion to PWV. The present findings therefore reinforce the notion that both AIx and PWV may be important cardiovascular measures in youth (Thurn et al. 2015).

It is increasingly recognized that the cardiometabolic benefits of MVPA are principally attributable to the vigorous element of this metric, despite this typically accounting for only a small proportion of a youth’s total daily physical activity (~ 4 min; Carson et al. 2014). In combination with the commonly cited barrier that youth do not have enough time to engage in sufficient physical activity, this has led to a growing interest in the potential efficacy of HIIT at improving the cardiometabolic health of youth, with the general consensus of recent systematic reviews and meta-analyses being that HIIT may be beneficial (Logan et al. 2014; Costigan et al. 2015; Eddolls et al. 2017). However, the majority of the studies underpinning these reviews have involved traditional exercise modalities, such as sprinting (running or cycling), the long-term sustainability of which is questionable (Weston et al. 2016). Furthermore, the majority have considered the influence of HIIT on individual markers of cardiometabolic risk despite the clustering of factors having been acknowledged for many years (Reaven 1988). A single factor is unlikely to define cardiometabolic health in youth (Stoner et al. 2017) and thus influencing individual factors does not necessarily translate to beneficial changes in the overall risk score. Indeed, in the current study, HIIT did not influence clustered cardiometabolic risk, irrespective of asthma status, despite being associated with improved blood pressure and lipids at mid-intervention. This lack of translation to global cardiometabolic benefit may be indicative that the changes were not sustained for a sufficient period, possibly due to insufficient progression within the HIIT protocol throughout the 6-month period, or that these factors are not the most influential in determining overall cardiometabolic health. With regards to those with asthma, it is possible that the parameters of cardiometabolic risk considered in this study are not those primarily effected by/involved in this condition; the key parameters largely remain to be elucidated and are likely dependent on numerous factors such as weight status, age, sex, and the specific asthma phenotype. Furthermore, future research should consider including a confirmation of asthma diagnosis to avoid potential confounding effects associated with the mis-diagnosis of asthma.

There is little consensus regarding the influence of exercise on arterial stiffness in youth. Ratgeber et al. (2015) similarly found no influence in a cross-sectional comparison of trained and untrained children, others have reported significantly lower arterial stiffness following HIIT (Chuensiri et al. 2018), circuit training (Watts et al. 2004), aerobic training (Meyer et al. 2006) or a combined aerobic and resistance training protocol (Farpour-Lambert et al. 2009). The variation in the training protocols and the use of flow-mediated dilation or PWV to assess arterial stiffness limit conclusions regarding the basis for these equivocal findings. Indeed, whilst both flow-mediated dilation and PWV provide insights to vascular function, it is important to note their different vascular physiological implications, with FMD reflecting nitric oxide bioavailability as a marker of endothelial health and increasing PWV reflecting adverse structural changes in the arterial wall. However, it is worthy of note that advantageous influences of exercise on arterial stiffness have only been reported in obese children (Chuensiri et al. 2018; Watts et al. 2004; Farpour-Lambert et al. 2009; Meyer et al. 2006). This may reflect the absence of derangements in vascular function in normal weight children, limiting the potential benefits that can be elicited through HIIT. Further research is nonetheless required to elucidate the potential efficacy of HIIT in overweight and obese youth with asthma, an increasingly common phenotype (Di Genova et al. 2018). Alternatively, the lack of beneficial effect in the present study may reflect an insufficient exercise intensity; whist the minimum exercise intensity required to elicit improvements remains to be elucidated, low-to-moderate intensity exercise has previously been reported to be ineffective at improving vascular function (Farpour-Lambert et al. 2009; Ashor et al. 2015).

As the longest HIIT intervention in children to date, and first to consider PWV and AIx, the present study extends our understanding of potential strategies to mitigate an increased cardiometabolic risk in youth, highlighting that those with mild-to-moderate asthma should be considered similarly to their non-asthma peers. Nonetheless, several potential limitations should be considered. First, the majority of the participants were Caucasian adolescents recruited from a predominately urban area; combined with the sample size these considerations may limit the generalizability of the findings. Furthermore, adiposity was estimated indirectly using common epidemiological measures, which could not distinguish the independent role of fat and fat-free mass in determining cardiometabolic risk and its response to HIIT. An important limitation of the current study was that the majority of those with asthma were characterized as mild and well controlled, potentially due to a self-selection bias; it is possible that cardiometabolic abnormalities would have been more apparent in those with more severe asthma. It is also important to highlight that self-reported GP diagnosis was used. However, the current range of disease severities is proportionally representative of the general asthma population. It is also pertinent to note, however, that asthma diagnoses in the current study were not verified using reversibility or other challenge tests which may result in the overdiagnosis of asthma. As with any long-term intervention study, poor adherence of some participants may have limited the overall influence of the intervention, but the current school-based delivery method increases the ecological validity of the findings rather than a contrived laboratory-based intervention.

## Conclusion

In conclusion, adolescents with controlled, mild asthma do not demonstrate any discernible differences in arterial stiffness compared to their healthy peers. Furthermore, neither arterial stiffness nor clustered cardiometabolic risk are influenced by a 6-month school-based HIIT intervention in adolescents. The present findings highlight that blood pressure, AIx and PWV should be considered physiologically distinct constructs that warrant further investigation as potential markers of cardiovascular health in children.

## Abbreviations

Aix: Augmentation index
FENO: Fraction of exhaled nitric oxide
FEV_1_: Forced expiratory volume in one second
FVC: Forced vital capacity
HIIT: High-intensity interval training
HDL: High-density lipoprotein
HR: Heart rate
LDL: Low-density lipoprotein
MVPA: Moderate-to-vigorous physical activity
PCA: Principal component analysis
PWA: Pulse wave analysis
PWV: Pulse wave velocity
Τ: Total cholesterol
TG: Triglycerides
UK: United Kingdom
